# Characterization of White Frost on Exocarpium Citri Grandis: Flavonoid Crystallization Enhances Anti-Inflammatory Activities

**DOI:** 10.3390/foods14244313

**Published:** 2025-12-15

**Authors:** Mengxue Yang, Wanbing Chen, Zhenjie Zeng, Pingzhi Wu, Hongqi Xia, Congyi Zhu, Ruoting Zhan, Jiwu Zeng

**Affiliations:** 1 Institute of Fruit Tree Research, Guangdong Academy of Agricultural Sciences, Key Laboratory of South Subtropical Fruit Biology and Genetic Resource Utilization, Ministry of Agriculture and Rural Affairs, Guangdong Provincial Key Laboratory of Science and Technology Research on Fruit Tree, Guangzhou 510640, China; mengxueyang0929@163.com (M.Y.); chenwanbing@gdaas.cn (W.C.); zhenjiezeng@163.com (Z.Z.); wupingzhi@gdaas.cn (P.W.); xiahongqi@gdaas.cn (H.X.); zhucongyi@gdaas.cn (C.Z.); 2 School of Pharmaceutical Sciences, Guangzhou University of Chinese Medicine, Guangzhou 510006, China

**Keywords:** *Exocarpium Citri Grandis*, naringin, *Citrus* processing, anti-inflammatory

## Abstract

*Exocarpium Citri Grandis* (ECG) is a distinctive medicinal and edible product originating from southern China and is often covered with a layer of characteristic “white frost” (WF). This study investigated the composition, formation mechanism, microbial safety, and anti-inflammatory activity of the WF. Multi-technique analyses revealed that WF mainly consisted of crystalline naringin (~80% of total mass). Drying-induced shrinkage and rupture of oil glands on ECG suggested metabolite migration and surface crystallization as the key mechanisms for WF formation. Microbial profiling revealed no significant differences in fungal and bacterial communities between WF and non-frost (NF) samples, and none of eight common mycotoxins was detected, confirming its microbial safety. Brewing tests demonstrated that water boiling for 30 min achieved efficient extraction of naringin, with higher yields in WF samples than in NF samples. In RAW264.7 cells, both WF and NF extract significantly inhibited lipopolysaccharide-induced NO production as well as the secretion and transcription of TNF-α, IL-6, IL-1β, iNOS, and NF-κB, with WF extract showing a stronger effect. Overall, these findings indicate that WF originates from endogenous flavonoid crystallization rather than microbial contamination and enhances the anti-inflammatory activity. This study provides a scientific basis for quality evaluation, processing optimization, and standardization of ECG products.

## 1. Introduction

*Exocarpium Citri Grandis* (ECG), locally known as Huajuhong, is derived from the nearly ripe fruits of the citrus variety *Citrus grandis* “Tomentosa” (commonly known as Huazhou pomelo) and is a distinctive medicinal and edible product originating from southern China. Its authentic production region is located in Huazhou City, Guangdong Province, and Huazhou-produced ECG was recognized as a National Geographical Indication Product of China in 2007 [1]. In 2024, ECG was officially included in the List of Substances with Both Medicinal and Edible Uses by the National Health Commission of China. Traditionally, ECG has been recorded for its potent efficacy in relieving cough and resolving phlegm and therefore has been widely used to alleviate respiratory symptoms. Recent pharmacological studies have demonstrated that ECG possesses diverse biological activities, such as antioxidant, anti-inflammatory, antimicrobial, and antitumor activities [2].

The pharmacological activities of ECG can be mainly attributed to flavonoids, particularly naringin, a principal compound contributing to its expectorant and anti-inflammatory effects [2], and polymethoxylated flavones further strengthen or contribute to its anti-inflammatory and lipid-regulating functions [3,4,5]. In addition, coumarins and terpenoids, despite their relatively low abundance, play supplementary roles in its ‘qi’ regulatory, antibacterial, and respiratory symptom-relieving activities, while polysaccharides mainly contribute to its auxiliary immunomodulatory and antioxidant effects [6,7]. Overall, these compounds together establish a multi-component biological basis, where primary and secondary metabolites jointly sustain the therapeutic activities of ECG [8]. The accumulation and stability of these metabolites are significantly influenced by various factors such as harvest time, varietal differences, and processing methods, which collectively determine the quality and bioactivity of ECG [9,10,11].

Traditionally, fresh fruits of Huazhou pomelo are harvested at the near-mature stage, when the diameter is about 6–8 cm, for balancing the yield and quality since the naringin content tends to decline during fruit development [12]. After harvesting, the fruits are washed and subjected to boiling in water for inactivation of the enzymes, followed by whole-fruit drying over a furnace at high temperature [13]. With advances in processing technologies, modern production of ECG often employs programmable electric ovens with multi-stage temperature control, which maintains the temperature at ~55 °C in the early phase and at ~65 °C in the later phase, with a total drying duration of about 36 h [14]. The obtained product is well accepted by consumers [15].

During processing, a conspicuous layer of “white frost” is often formed on the surface of the final product of ECG. While a thin and uniform layer of frost is traditionally regarded as a sign of potency, uneven or granular deposits of “white frost” on the surface tend to cause disputes on its quality and concerns on its safety. A survey of Huazhou enterprises has revealed that in some batches of production, more than 30% of ECG products exhibit visible frosts (Table S1, Supplementary Materials). Thus, it is essential to fully characterize the formed “white frost” to provide a scientific basis for quality evaluation and standardization of ECG products.

This study systematically investigated the “white frost” on the surface of ECG. Microstructural changes during processing and in the final product were examined by electron microscopy and histological sectioning, while the chemical composition was characterized using energy dispersive spectroscopy (EDS), infrared spectroscopy, and UPLC/Q-TOF. The microbial safety was assessed through plate counting, ITS sequencing, and mycotoxin detection, while the anti-inflammatory activities of samples with and without frost were evaluated in a RAW 264.7 macrophage model. The findings will help elucidate the constituents, formation mechanism, safety, and pharmacological relevance of the “white frost”, thereby providing a scientific basis for quality evaluation, processing optimization, and standardization of ECG products.

## 2. Materials and Methods

### 2.1. Experimental Materials

White-frost (WF) and non-frost (NF) ECG samples were provided by Guangdong Huazhou Juxing Huajuhong Group Co., Ltd. (Maoming, China). The drying process of *Citrus grandis* “Tomentosa” was carried out strictly in accordance with the Maoming local standard DB4409/T32-2023 [16], Code of practice for drying processing of Citri Grandis Exocarpium. Fresh fruits were washed, drained, and subjected to high-temperature fixation at 100–105 °C for 5–8 min under hot air, followed by hot-air drying at 55–70 °C for 96 h in a forced-air oven. Temperature and air flow were continuously monitored to ensure stable drying conditions and processing consistency. Samples for microscopic sectioning and histological staining were collected at 0 h and 12 h of the drying process, whereas samples for microbial enumeration were collected at 0 h, 24 h, 48 h, 72 h, and 96 h. Unless otherwise specified, all subsequent experiments were performed using the final dried products, which were cooled in a dry, well-ventilated environment and kept unpackaged to prevent moisture reabsorption before analysis.

RAW264.7 cells routinely cultured in DMEM medium containing 10% fetal bovine serum (FBS) and 1% penicillin-streptomycin, in a 5% CO_2_ incubator at 37 °C with 95% air. All cells and culture media were obtained from Procell Life Science & Technology Co., Ltd. (Wuhan, China). For reagents, naringin (HPLC purity > 98%) was purchased from Yuanye Biotechnology Co., Ltd. (Shanghai China); AxyPrep DNA Gel Extraction Kit was obtained from AXYGEN Inc. (Union City, CA, USA); TransStart Fastpfu DNA Polymerase was obtained from TransGen Biotech (Beijing, China); CCK-8 reagent was provided by Lanjieke Technology Co., Ltd. (Shanghai, China); ELISA detection kits for TNF-α, IL-6, iNOS, and IL-1β were purchased from Huding Biotechnology Co., Ltd. (Shanghai, China); NO detection kit, BCA Protein Quantification kit, protease inhibitor, and phosphatase inhibitor were obtained from Beyotime Biotechnology Co., Ltd. (Shanghai, China). SteadyPure Rapid RNA Extraction Kit, Evo M-MLV Reverse Transcription Premix Kit (with gDNA removal reagent, for qPCR) Ver.2, and SYBR Green Pro Taq HS Premix qPCR Kit (with ROX) were purchased from Hunan Accurate Biotechnology Co., Ltd. (Changsha, China).

### 2.2. Experimental Methods

#### 2.2.1. Microscopic and Morphological Observation

Fresh ECG, NF obtained after 12 h of drying, and WF obtained after 12 h of drying were selected for histological staining. Based on methods modified from previous reports [17], small pieces of pericarp were fixed in FAA solution, dehydrated through a graded ethanol series, embedded in paraffin, and sectioned at 8–10 μm. The sections were stained with safranin and then fast green, dehydrated, cleared, and mounted with neutral resin. Structural differences among the three samples were observed and photographed under a light microscope.

The surface frost morphology was examined using stereomicroscopy and scanning electron microscopy (SEM). Based on methods modified from previous reports [18], a 2 × 2 cm piece of pericarp was placed on a stereomicroscope stage for surface observation, while surface deposits were gently collected with a cotton swab, mounted on conductive adhesive, sputter-coated with gold, and imaged under SEM at magnifications of 200×, 500×, and 2000×. Frost powder scraped from the surface, together with naringin reference standard, was similarly mounted, coated, and observed at 2000×. Representative images were recorded for morphological comparison. Elemental composition of the white frost powder was analyzed by SEM equipped with an EDS detector. The representative spectra and elemental maps were collected to identify and quantify the major elements.

#### 2.2.2. Component Analysis

The infrared spectra of the white frost powder on the surface of ECG were obtained using the potassium bromide (KBr) pellet method [19]. Frost powder was finely ground with KBr, pressed into pellets, and scanned across the range of 400–4000 cm^−1^ to identify the characteristic absorption bands.

For qualitative analysis [19], scraped frost samples were washed with 3 mL of 50% methanol, adjusted to a final volume of 5 mL, and filtered through a 0.22 μm microporous membrane. Analyses were carried out on a Thermo Scientific Accucore C18 column (100 mm × 2.1 mm, 2.6 μm). The mobile phases were 0.1% formic acid aqueous solution (A) and methanol-acetonitrile (B), with the gradient of 0–2 min, 90% A; 10 min, 80% A; 20 min, 20% A; 25 min, 0% A; re-equilibration at 90% A from 25.5 min. The flow rate was 0.4 mL/min; the column temperature was 35 °C; and the injection volume was 2 μL. Mass spectrometry was performed with an electrospray ionization (ESI) source in the positive ion mode under the following conditions: nebulizer pressure 35 psi, capillary voltage 3.5 kV, drying gas temperature 320 °C (8 L/min), sheath gas temperature 350 °C (11 L/min), ion transfer tube 320 °C, and a scanning range of m/z 100–1700.

For quantitative determination of naringin, samples were prepared as described for UPLC-DAD [20]. Naringin reference standards were dissolved in 80% methanol to prepare calibration solutions with concentrations ranging from 31.25 to 500 μg/mL. HPLC analysis was performed on a Hypersil GOLD C4 column (2.1 mm × 100 mm, 1.9 μm) with mobile phase A (0.1% formic acid solution) and mobile phase B (acetonitrile). The gradient program was 0–5 min, 90% A; 8 min, 60% A; 12 min, 45% A; 25 min, 10% A; followed by 5 min re-equilibration at 90% A. The flow rate was 0.3 mL/min; the column temperature was 30 °C; the injection volume was 2 μL; and detection was carried out at 284 nm.

#### 2.2.3. Microbial and Mycotoxin Detection

The mycotoxins in ECG were detected using standardized AOAC methods. Specifically, aflatoxin B_1_, aflatoxin B_2_, and total aflatoxins were analyzed according to AOAC Official Method 2008.02 (2008) [21]. Patulin was determined following AOAC 2000.02 (2004) [22]. Ochratoxin A was detected using AOAC 991.44 (1996, reaffirmed 2002) [23]. Zearalenone was analyzed in accordance with AOAC 985.18 (1988, reaffirmed 2002) [24]. Deoxynivalenol (vomitoxin) was determined using AOAC 986.18 (1990) [25]. Fumonisin B_2_ was analyzed according to AOAC 2001.04 (2001) [26].

During the drying process of ECG, WF and NF samples were collected from the same drying chamber at 12 h intervals. The total aerobic bacterial count and total fungal count were determined by the plate count method according to the general rules of the Chinese Pharmacopoeia.

Sterile disposable swabs dipped in physiological saline were used to wipe the surface of ECG. Genomic DNA was extracted from the washing solution using the cetyltrimethylammonium bromide (CTAB) method [27,28]. Briefly, samples were incubated with CTAB extraction buffer at 65 °C for 30 min, followed by extraction with chloroform: isoamyl alcohol (24:1, *v*/*v*). DNA was then precipitated with isopropanol, washed with 70% ethanol, and finally resuspended in TE buffer. The extracted genomic DNA was used as the template for PCR amplification. Bacterial 16S rDNA (V3–V4 region) and fungal ITS1 regions were amplified using barcode-tagged primer pairs 338F/806R and ITS1F/ITS2R, respectively [29] (primer sequences are listed in Table S2, Supplementary Materials). PCR products were verified by electrophoresis on 2% agarose gels, and equimolar amounts were pooled for downstream analysis. Target fragments were purified by gel extraction, and sequencing libraries were prepared with the TruSeq DNA PCR-Free Sample Preparation Kit. The libraries were quantified by Qubit and qPCR, and sequenced on an Illumina HiSeq2500 platform in a paired-end 250 bp (PE250) mode after quality control.

#### 2.2.4. Naringin Contents in ECG Decoctions Obtained with Different Brewing Methods

Different brewing preparations of ECG decoctions were prepared following previously published methods [30,31], with slight modifications. Briefly, the whole WF and NF fruits were cut into pieces of approximately 10 g each and extracted with different solvents at a ratio of 1 g: 100 mL. Brewing was conducted with the following methods. For hot water soaking (HW): hot ultrapure water (100 °C) was added to the samples (ambient temperature 25 °C) and maintained for 30 min. For boiling water decoction (BW): ultrapure water at 100 °C was added and the samples were continuously boiled for 30 min (heating power: 300 W). For ethanol soaking, the samples were soaked in 15% (LE) or 50% ethanol (HE), respectively, at room temperature (25 °C) for 7 days. The extraction yield of naringin was calculated using Equation (1):

(1) $$\mathrm{Decoction}\;(\%)=\frac{m_{decoction}}{m_{raw\;ECG}}\times 100\%$$

where *m_decoction_* is the mass of naringin in the decoction, *m_raw ECG_* is the mass of naringin in the raw ECG.

The decoctions obtained by the above methods were collected and adjusted to the initial volume. Quantitative determination of naringin content (ug Naringin/mL decoction) was performed by UPLC-DAD, and the detection conditions were the same as described in Section 2.2.2.

#### 2.2.5. Cell-Based Assays of Anti-Inflammatory Activity

The whole fruits of WF and NF were cut into large chunks and subjected to boiling for 30 min. The decoctions (1000 mL each) were concentrated by rotary evaporation at 50 °C and lyophilized. The yields of the concentrate were 2.97 g/10 g for NF and 3.77 g/10 g for WF. For stock solutions, 200 mg of each extract was dissolved in 2 mL of 50% DMSO, vortexed until complete dissolution, sterilized by filtration (0.22 μm), and then stored at 4 °C.

RAW264.7 cells were seeded in 96-well plates at 1 × 10^5^ cells/mL (100 μL/well). The experimental groups included the blank (medium only), control (untreated cells), and treatment groups with six concentrations of NF or WF extracts (15.625, 31.25, 62.5, 125, 250, and 500 μg/mL, with six replicates each). After 24 h of incubation, 100 μL of CCK-8 reagent was added to each well, followed by incubation for 4 h in the dark. The absorbance was measured at 450 nm. The cell survival rate was determined according to Equation (2).

(2) $$\mathrm{Cell}\;\mathrm{viability}\;(\%)=\frac{\left(A_{experimental}-A_{blank}\right)}{\left(A_{control}-A_{blank}\right)}\times 100\%$$

where the *A_experimental_*, *A_blank_*, and *A_control_* denote the absorbance at 540 nm of experimental, blank, and control group, respectively.

RAW264.7 cells in logarithmic growth were seeded into 6-well plates at 3 × 10^5^ cells/mL (2 mL/well) and cultured for 24 h. The experimental groups included the blank (medium only), model (1 μg/mL LPS treated), and treatment groups with NF or WF extracts at 50, 100, and 200 μg/mL combined with 1 μg/mL LPS. Extracts were added 30 min before LPS stimulation. After 24 h, cells were centrifuged at 1000 rpm for 10 min to separate the supernatant and pellet. The supernatants were collected for the determination of nitric oxide (NO) levels using a commercial NO detection kit, according to the manufacturer’s instructions. The cell pellets were lysed with a protein extraction kit to obtain total protein. The concentrations of TNF-α, IL-6, IL-1β, and iNOS were subsequently quantified using commercial ELISA kits.

Total RNA was extracted with the SteadyPure Rapid RNA Extraction Kit and reverse transcribed into cDNA. β-actin was used as the reference gene. Primer sequences are listed in Table S2. qRT-PCR was performed using SYBR Green Pro Taq HS Premix under the following conditions: 95 °C for 30 s, followed by 40 cycles of 95 °C for 5 s and 60 °C for 30 s. Gene expression of *TNF-α*, *IL-6*, *iNOS*, *IL-1β*, and *NF-κB* was analyzed by the 2^−ΔΔCt^ method.

### 2.3. Data Analysis

The obtained data were statistically analyzed and plotted using SPSS Statistics 27 software and GraphPad Prism 8.0.2 software. Results were expressed as mean ± standard deviation (x ± s). Data normality was assessed prior to analysis. Group differences were evaluated using one-way ANOVA, followed by Tukey’s HSD post hoc test for multiple comparisons when appropriate. All experiments were performed in triplicate.

## 3. Results and Discussion

### 3.1. Microstructural and Compositional Characterization of White-Frost ECG

**Figure 1:**
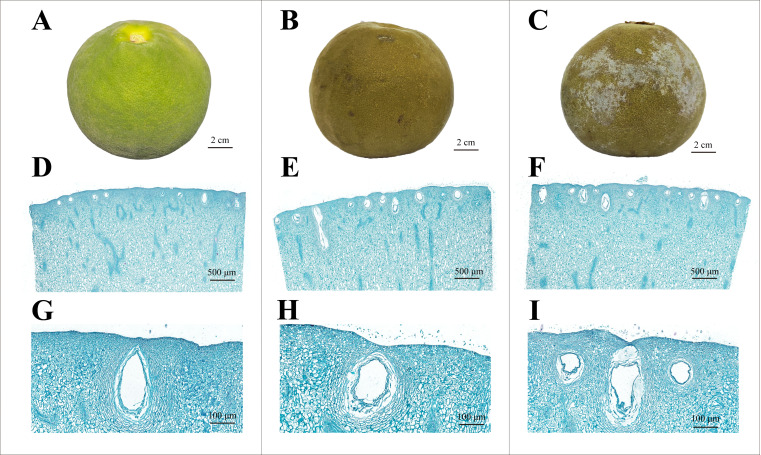
**Safranin** O-fast green staining of the surface of ECG. Macroscopic appearance of fresh ECG (**A**), NF obtained after 12 h of drying without frost (**B**), and WF obtained after 12 h of drying with surface frost (**C**). (**D**–**F**) Corresponding sections stained with safranin O-fast green at ×2 magnification. (**G**–**I**) Corresponding stained sections at ×20 magnification.

Figure 1A–C shows the images of fresh ECG, NF obtained after 12 h of drying, and WF obtained after 12 h of drying. WF ECG, unlike fresh ECG which was bright green, showed small continuous patches of white frost on the surface. Microscopic observation revealed pronounced structural changes in the pericarp under high temperature treatments. Fresh ECG fruit exhibited exocarp cells tightly arranged in a regular palisade pattern, with relatively uniform sizes and multiple epidermal layers (Figure 1D). The oil glands appeared well-developed, and were surrounded by intact cavities, epidermal cells, and sheath cells (Figure 1G). After 12 h of drying, the pericarp of NF exhibited less distinct stratification, with enlarged intercellular spaces and slightly shrunken oil glands with blurred boundaries, though the epidermal cells surrounding the oil glands remained intact (Figure 1E,H). In contrast, WF displayed decreases in cell uniformity and weakening of reticular connections, with marked invagination expansion (Figure 1F). Oil glands were further shrunken and deformed with indistinct boundaries, accompanied by collapse of adjacent epidermal cells (Figure 1I). These observations provided direct histological evidence for the formation of the “white frost” on ECG surface.

Previous studies have shown that the compact arrangement of exocarp cells in fresh citrus fruit is conducive to the maintenance of tissue integrity and reduction in water and gas loss [32], whereas drying can induce significant microstructural changes, including cell shrinkage and deformation and intercellular space expansion. SEM has also revealed that drying leads to denser pericarp tissues with reduced porosity, which is in sharp contrast with the plump morphology of fresh fruits [33]. The formation of “white frost” on ECG is probably associated with the disruption of the integrity of internal cell structures, particularly oil glands, which are critical reservoirs of secondary metabolites and play essential roles in fruit quality and defense [34]. Previous studies have associated postharvest physiological disorders such as “segmental drying” with cell wall degradation, water transport imbalance, and lignin accumulation, all of which are related to pericarp tissue deterioration [35,36,37,38]. Collectively, these results suggested that the deterioration of pericarp tissues during drying is closely associated with the formation of “white frost” on ECG, where pericarp invagination and oil glands rupture represent the key structural basis for its formation.

**Figure 2:**
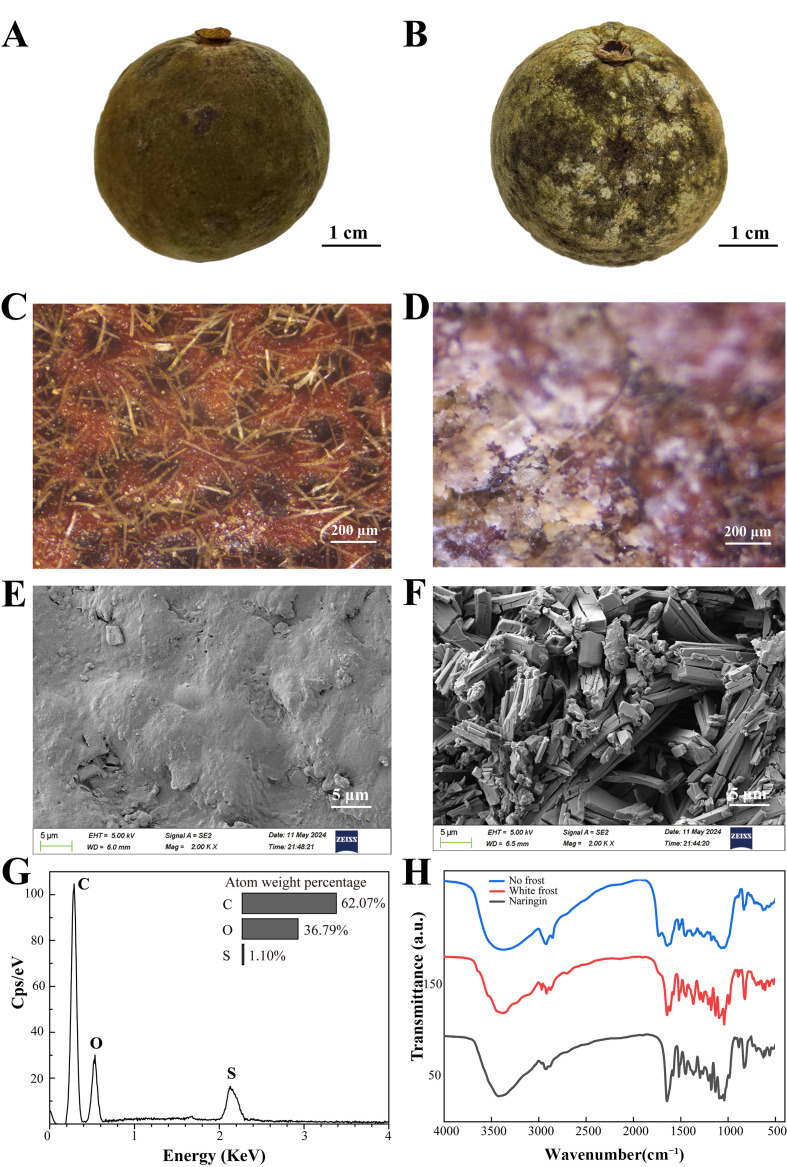
Surface morphology and component analysis of ECG. Macroscopic appearance of NF (**A**) and WF (**B**). Stereomicroscopic images of NF (**C**) and WF (**D**). Scanning electron microscopy (SEM) images of NF (**E**) and WF (**F**). (**G**) Energy-dispersive X-ray spectroscopy (EDS) map of surface crystals from WF. (**H**) Fourier-transform infrared (FTIR) spectra of naringin standard and surface powders from NF and WF.

As shown in Figure 2A,B, NF presented a smooth and clean surface, while WF was characterized by irregular patches of frost on the surface. Under stereomicroscopy (Figure 2C,D), NF displayed bright and well-defined oil glands, whereas WF was uniformly covered by a layer of frost, with obviously smaller and lighter oil glands. SEM (Figure 2E,F) further revealed that NF had a relatively smooth surface characterized by evenly distributed convex wax and recessed stomata, while WF showed a surface densely covered by acicular and block-like crystals. These crystals were aggregated into elongated clusters with regular morphologies, which measured approximately 2–4 μm in diameter and 20–30 μm in length. EDS analysis of the frost crystals (Figure 2G) showed that they were mainly composed of C and O, with a small amount of S. FTIR spectroscopy (Figure 2H) detected significant characteristic peaks, including a broad phenolic hydroxyl (-OH) band at 3500 cm^−1^, a conjugated carbonyl (C=O) peak at 1664 cm^−1^, and aromatic ring vibration peaks at 1600/1512 cm^−1^ [39]. These peaks exhibited more than 95% similarities to those in the spectrum of the naringin reference standard. Naringin-specific peaks were identified in both WF and NF samples; however, the WF exhibited more pronounced transmittance changes and sharper peak shapes, indicating that it has a higher naringin content.

### 3.2. Microbial Dynamics and Safety Evaluation of White-Frost ECG

The major postharvest diseases of citrus—such as green mold caused by *Penicillium digitatum*, blue mold caused by *Penicillium italicum*, and sour rot caused by *Geotrichum candidum*—typically produce white or grayish-green frost-like deposits on the fruit surface [40]. These symptoms closely resemble the white frost observed on ECG. Accordingly, ITS1 amplicon sequencing was conducted to compare the fungal communities present on the surfaces of WF and NF samples.

**Figure 3:**
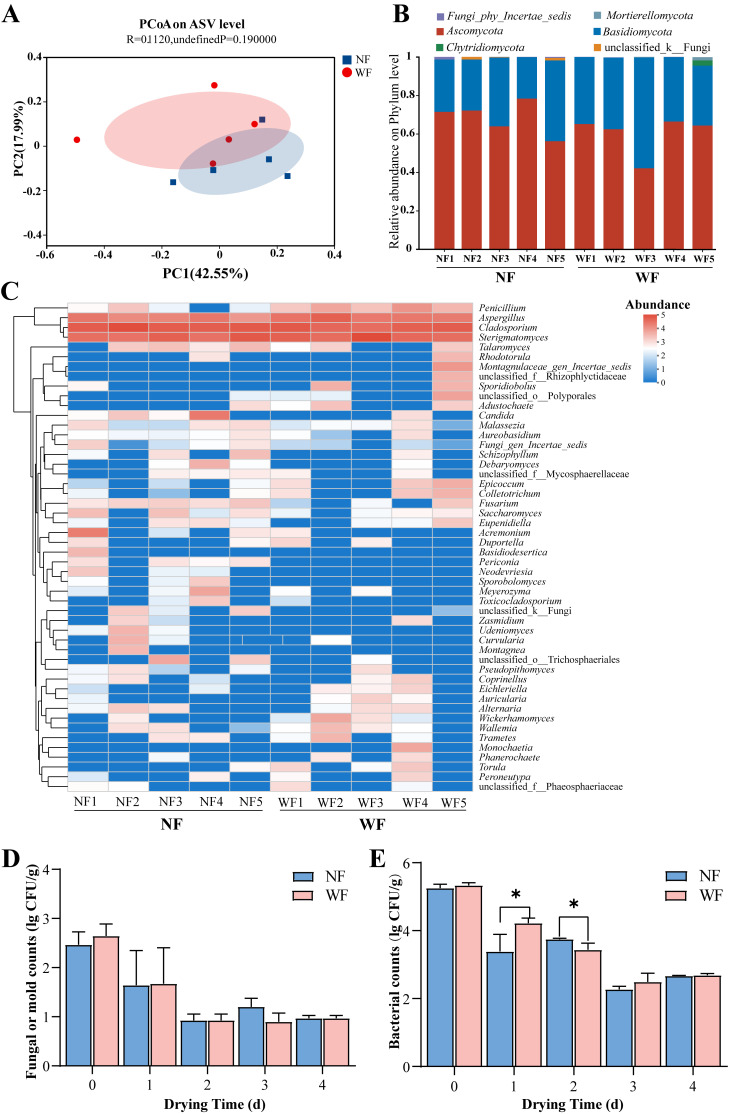
Analysis of surface microorganisms on ECG. (**A**–**C**) ITS-based fungal diversity analysis: (**A**) Fungal composition at the genus level; (**B**) Principal coordinate analysis (PCoA) at the amplicon sequence variant (ASV) level based on Bray–Curtis distances; (**C**) Fungal community composition at the phylum level (top 50 taxa in relative abundance). (**D**,**E**) Changes in microbial populations in WF and NF: (**D**) fungal or mold counts and (**E**) bacterial counts. * indicates highly significant differences (*p* < 0.05).

Fungal community sequencing of multiple batches of ECG samples generated 923,299 valid fungal sequences after quality control. Clustering at the 97% sequence similarity generated a total of 701 amplicon sequence variants (ASVs, ranging from 60 to 164 for different samples), providing a basis for community structure analysis. Principal coordinate analysis (PCoA) at the ASV level (Figure 3A) revealed that the WF and NF samples had no significant difference in fungal composition, suggesting that they have similar core community structures. At the phylum level (Figure 3B), Ascomycota was the dominant phylum (>80% relative abundance) in both WF and NF, followed by Basidiomycota, which is consistent with the general dominance pattern observed in citrus medicinal plants.

Heatmap analysis at the genus level (Figure 3C) further indicated high degrees of similarity in fungal composition between WF and NF samples, where *Penicillium*, *Aspergillus*, *Cladosporium*, *Fusarium*, and *Alternaria* were identified as the core genera. This is consistent with the reported stability of fungal communities on medicinal plant surfaces [41]. The widespread presence of *Cladosporium* and *Alternaria* reflects their strong environmental adaptability, while the detection of *Fusarium* may be linked to warehouse contamination [42,43]. In addition, the presence of *Penicillium* and *Aspergillus* suggested that ECG is susceptible to saprophytic fungal infection during storage [44]. Overall, the similarities in fungal community structure indicated that white frost formation on ECG is not associated with fungal invasion.

Similarly to the fungal findings, β-diversity analysis showed no significant differences between the bacterial communities of WF and NF (Figure S1A in Supplementary Materials). The major bacterial taxa at both the phylum and genus levels also showed highly consistent relative abundances between the two groups (Figure S2B,C in Supplementary Materials), suggesting that the presence of white frost is not associated with bacterial invasion.

During drying, the microbial count declined regularly with the extension of drying time. Both the fungal or mold counts (Figure 3D) and bacterial counts (Figure 3E) showed significant decreases, which could be attributed to water activity reduction caused by dehydration and microbial inactivation caused by thermal effects [45,46]. WF and NF (Figure 3D,E) exhibited consistent trends in total colony, mold, and yeast counts, with no statistically significant difference (*p* > 0.05), suggesting that both WF and NF experienced comparable microbial dynamics during drying.

Given that fungal contamination and associated mycotoxins represent critical safety risks for medicinal materials and dried foods, eight common mycotoxins (aflatoxin B_1_, B_2_, total aflatoxins, patulin, ochratoxin A, zearalenone, deoxynivalenol, and fumonisin B_2_) were analyzed in WF and NF samples. As a result, none of the target toxins was detected (Table S3, Supplementary Materials), which is consistent with the reports of generally low contamination rates in medicinal materials and agricultural products. A global systematic survey has indicated that the detection rate of aflatoxins was only 6.0% in medicinal plants (concentrations ranging from 0.01 to 19.70 µg/kg) [47]. The absence of toxins in WF and NF may be related to the inhibitory effects of specific secondary metabolites in ECG (such as limonene) on toxin-producing fungi [48,49].

In summary, the results from fungal community profiling, bacterial community analysis, microbial count dynamics, and mycotoxin detection collectively demonstrate that the white frost on ECG is not caused by fungal or bacterial invasion. Instead, it is a physicochemical phenomenon primarily resulting from the crystallization of flavonoids during high-temperature drying.

### 3.3. Identification and Quantification of Components in ECG White Frost

A UPLC/Q-TOF analysis was performed on the surface powders collected from WF and NF samples. The chromatograms obtained from the UPLC/Q-TOF analyses are provided in Figures S2 and S3 in Supplementary Materials. As a result (Table 1), a total of 12 characteristic compounds were identified, including eight flavonoids (luteolin-β-rutinoside, naringenin, rhoifolin, naringenin-O-glucoside, dimethoxyflavone, isovitexin, naringin, and diosmin), one triterpenoid (limonin), one organic acid (quinic acid), and two lipids (isopalmitic acid and lauryl hydrogen sulfate). This compositional profile is consistent with previous reports on citrus-derived medicinal materials [50,51], indicating that the “white frost” is chemically complex but is predominantly composed of flavonoids. A semi-quantitative comparison of peak intensities further revealed that WF had markedly higher levels of naringin, naringenin, diosmin, and naringenin-7-O-glucoside, particularly naringin, which was nearly 20-fold that in NF.

**Figure 4:**
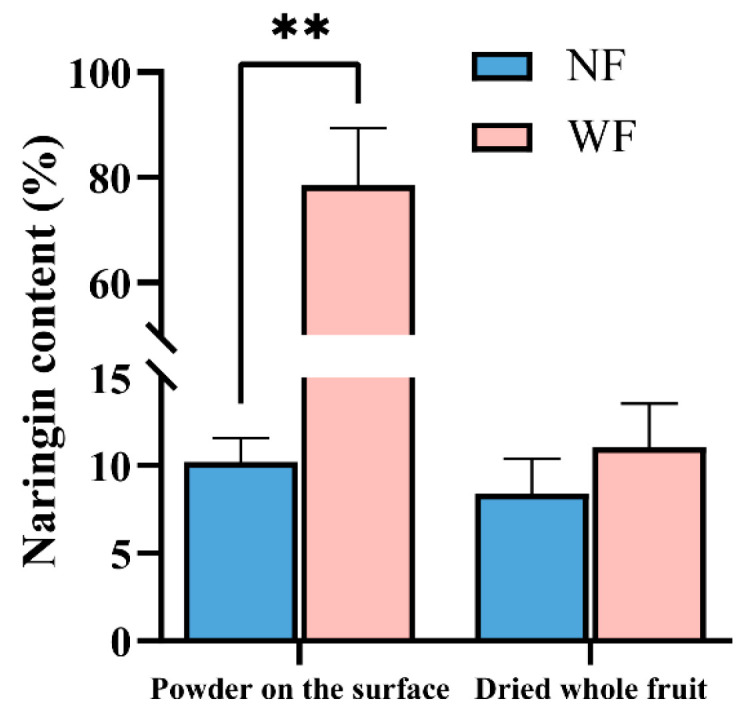
Naringin contents in the surface powder and whole fruit of WF and NF. ** indicates highly significant differences (*p* < 0.01).

To validate the above results, an HPLC analysis was performed (the chromatograms are shown in Figure S4 in Supplementary Materials). The results (Figure 4) showed that the naringin content in the surface powders of WF was 7.69 times of that in NF (*p* < 0.01), while no significant difference was observed in the total naringin level of the whole fruit between WF and NF (*p* > 0.05). FTIR spectra further confirmed this result, as WF surface powders displayed sharper and more intense absorption peaks specific to naringin, suggesting higher purity of naringin on WF surface. Taken together, these results indicated that naringin, which accounted for approximately 80% of the total crystalline mass, is the principal component of the “white frost”.

Mechanistically, the formation of “white frost” primarily results from the migration, enrichment, and eventual surface crystallization of endogenous flavonoids during the drying process. Naringin was identified as the major component of the frost, confirming that the crystalline layer originates from endogenous metabolites rather than external contamination. As a flavanone glycoside with low water solubility, naringin is predominantly stored in the albedo and oil glands of the peel [52,53]. During storage and drying, moisture loss generates a concentration gradient that promotes the outward diffusion of soluble constituents. Meanwhile, thermal processing or prolonged aging may partially disrupt oil gland structures and increase the permeability of the peel surface [54,55], thereby facilitating the migration of naringin toward the exterior. Once local supersaturation is reached, naringin crystallizes and deposits on the peel surface as a frost-like layer visible to the naked eye.

### 3.4. Extraction Rates of Naringin from WF and NF by Different Brewing Methods

**Figure 5:**
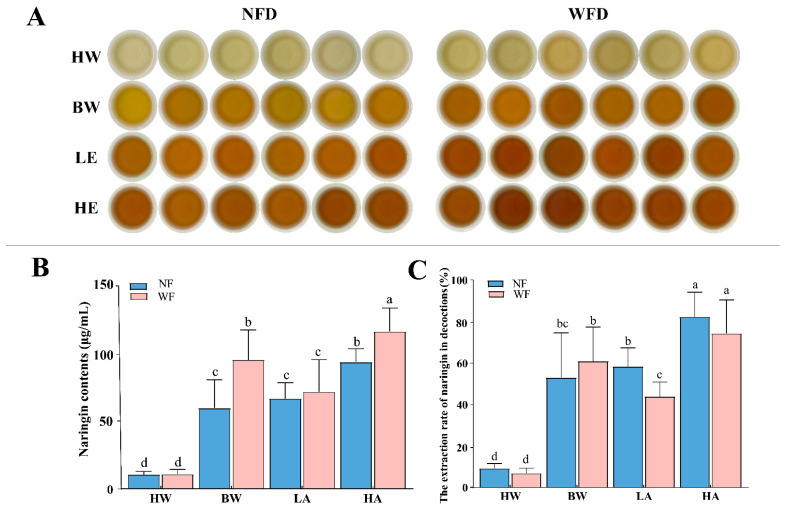
Effects of different brewing methods on the naringin content and extraction rate in WF and NF decoctions (WFD and NFD). (**A**) Appearance of decoctions prepared by different brewing methods; (**B**,**C**) Naringin content (**B**) and extraction rate (**C**) of WFD and NFD prepared by different brewing methods. HW, hot water soaking for 30 min; BW, boiling water decoction for 30 min; LE, soaking in 15% ethanol for 7 d; HE, soaking in 50% ethanol for 7 d. Different letters indicate significant differences (*p* < 0.05) among treatments.

HPLC was employed to determine the naringin content in WF decoctions (WFD) and NF decoctions (NFD) prepared with four brewing methods, including hot water soaking (HW), boiling water decoction (BW), 15% ethanol soaking (LE), and 50% ethanol soaking (HE). As shown in Figure 5, BW and HE resulted in obviously higher extraction rates of naringin. Considering the potential health risks associated with residual ethanol, BW was identified as a more suitable brewing method for ECG.

Importantly, regardless of the brewing methods, WFD had consistently higher naringin contents than NFD (*p* < 0.05). This phenomenon can be ascribed to two reasons. On the one hand, the crystalline naringin precipitated on WF surface is directly exposed to the solvent and is more readily dissolved, which increases the initial dissolution rate [56]. On the other hand, structural damage in WF tissues, particularly in oil glands, enhances the porosity and facilitates the outward migration of internal metabolites, thereby improving solvent penetration and component release [57]. These factors may together contribute to the superior extraction rate of naringin from WFD than that from NFD.

These results are consistent with previous findings that brewing conditions, such as temperature, duration, and solvent type, have strong impacts on the dissolution kinetics and bioavailability of active components in herbal teas and traditional Chinese medicines [58]. In the case of ECG, boiling water not only ensures sufficient extraction of flavonoid glycosides such as naringin but also avoids the safety risks associated with ethanol-based extraction. Therefore, water boiling for 30 min may be recommended as a standardized brewing method for maximizing the efficacy and safety in ECG consumption.

### 3.5. Effects of WF and NF Decoctions on Raw264.7 Cell Viability and Inflammatory Responses

**Figure 6:**
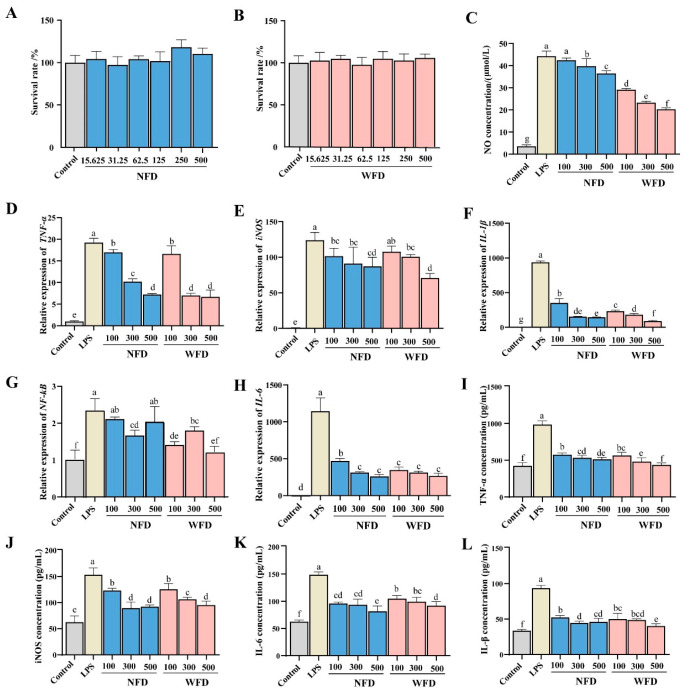
Effects of WF and NF decoctions (WFD and NFD) on the viability and inflammatory responses of RAW264.7 cells. (**A**,**B**) RAW264.7 cell viability; (**C**) NO secretion; (**D**–**H**) gene expression of *TNF-α*, *iNOS*, *IL-1β*, *NF-κB*, and *IL-6*; (**I**–**L**) protein/secretion levels of TNF-α, iNOS, IL-1β, and IL-6; The concentrations of NFD and WFD was expressed in units of µg/mL. Different letters indicate significant differences (*p* > 0.05).

Cytotoxicity assays demonstrated that both WFD and NFD showed no inhibitory effect on RAW264.7 cell viability across the tested concentrations (15.625, 31.25, 62.5, 125, 250, and 500 μg/mL), where cell viability consistently remained close to 100% (Figure 6A,B). In the LPS-induced inflammatory model with markedly elevated NO levels, both WFD and NFD significantly reduced the NO production in a dose-dependent manner (*p* < 0.05), with WFD showing a more pronounced inhibitory effect (Figure 6C).

ELISA results further revealed that LPS stimulation significantly increased the secretion of TNF-α, IL-6, and IL-1β, while treatments with medium and high doses of WFD and NFD significantly reduced these cytokines (*p* < 0.05), with WFD exerting superior inhibitory effects on TNF-α and IL-1β (Figure 6I–L). At the transcriptional level, qRT-PCR analysis showed that both WFD and NFD significantly downregulated the expression of *IL-6* and *IL-1β* (*p* < 0.05), along with reduced expression of *TNF-α*, *iNOS*, and *NF-κB* (Figure 6D–H). WFD again exhibited stronger inhibitory effects on *TNF-α* and *IL-1β* expression compared with NFD. These results indicate that within the non-cytotoxic concentration range, both WFD and NFD can alleviate LPS-induced inflammatory responses in RAW264.7 macrophages by inhibiting NO release, cytokine secretion, and pro-inflammatory gene expression, particularly WFD.

The anti-inflammatory activities of both WFD and NFD appear to involve multiple coordinated mechanisms. Both extracts inhibited NO production in a dose-dependent manner and significantly reduced the transcription and secretion of key pro-inflammatory cytokines (TNF-α, IL-1β, IL-6), while also suppressing NF-κB activation, thereby alleviating LPS-induced inflammatory responses [59,60,61]. In all assays, WFD exhibited a more pronounced inhibitory effect than NFD. This enhanced activity is likely related to the higher naringin content in WF. Naringin has been widely reported to exert antioxidant, anti-inflammatory, and metabolic regulatory effects [62,63], primarily through activation of the Nrf2 antioxidant pathway and inhibition of NF-κB signaling, leading to reduced cytokine expression and improved cellular protection [64]. It has also shown protective effects in various disease models, including organ injury, cardiometabolic disorders, and neurodegeneration [65,66]. Therefore, the higher naringin concentration in WF may be a key factor contributing to its stronger pharmacological effects, although further in vivo studies are needed to confirm its bioactivity and metabolic profiles.

## 4. Conclusions

This study systematically investigated the surface “white frost” of ECG through microstructural observation, component identification, microbial safety evaluation, and bioactivity assays. Microscopic and histological analyses revealed that the formation of “white frost” is closely associated with pericarp invagination and oil gland rupture, which facilitates the outward migration and crystallization of internal metabolites. SEM, EDS, and FTIR analyses demonstrated that the frost exhibited a crystalline structure primarily composed of high-purity naringin, along with polymethoxyflavonoids and trace amounts of non-flavonoid compounds. UPLC/Q-TOF and HPLC further confirmed that naringin is the predominant constituent, accounting for nearly 80% of the frost mass.

Microbial community sequencing and mycotoxin detection indicated that the formation of white frost is not associated with microbial contamination, as the WF and NF samples exhibited highly similar fungal and bacterial community structure, and no common mycotoxins were detected. Brewing experiments showed that the dissolution rate of naringin was significantly higher in WF samples, and boiling in water for 30 min was identified as the most effective and safe brewing method. Moreover, cellular assays demonstrated that both NF and WF are non-toxic to RAW264.7 cells and exhibit anti-inflammatory activities by inhibiting NO release and downregulating the expression and secretion of TNF-α, IL-1β, IL-6, iNOS, and NF-κB, with WF showing more pronounced effects.

The “white frost” on ECG represents the crystallization of pharmacologically active components. Its formation is closely related to processing-induced structural changes and metabolite migration and may enhance the bioactivity of ECG. These findings provide a scientific basis for quality evaluation, standardization, and rational utilization of ECG products in both medicinal and functional food applications.

## Figures and Tables

**Table 1:** UPLC/Q-TOF analysis of WF and NF surface powders.

No.	Name	Formula	Ionization Model	Precursor Ion (m/z)	Retention Time (min)	Error (ppm)	MS^2^ Fragment	Peak Height (×10^5^)	
								WF	NF
1	Luteolin-β-rutinoside	C_27_H_30_O_15_	M + H	595.1640	4.679	2.94	287.0549	1.64	ND
2	(±)-Naringenin	C_15_H_12_O_5_	M + H	273.0748	4.996	3.49	153.0182; 67.0181; 91.0544; 119.0490	8.33	5.82
3	Rhoifolin	C_27_H_30_O_14_	M + H	579.1691	5.018	3.00	271.0593; 85.0278	3.26	14.50
4	Naringenin-O-glucoside	C_21_H_22_O_10_	M + H	435.1274	5.025	2.70	273.0749; 153.0173; 195.0281; 399.10	6.68	1.69
5	Dimethoxyflavone	C_17_H_14_O_4_	M + H	283.0947	7.779	6.33	84.9594; 242.1069; 203.0323	1.62	1.63
6	Limonin	C_26_H_30_O_8_	M + H	471.1989	7.951	5.20	161.0554; 425.1925; 95.0090	4.06	ND
7	Quinic acid	C_7_H_12_O_6_	M − H	191.0575	0.586	−7.23	185.0301; 59.0145; 93.0345; 127.0405	4.38	4.93
8	Isovitexin	C_21_H_20_O_10_	M − H	431.0983	4.495	0.16	311.0573; 283.0619; 341.0666; 117.03	ND	1.57
9	Naringin	C_27_H_32_O_14_	M − H	579.1741	4.901	−3.74	271.062; 151.0039; 459.1178	202.89	10.10
10	Diosmin	C_28_H_32_O_15_	M − H	607.1666	5.147	0.40	299.056; 151.0020; 255.0269	2.33	ND
11	Isopalmitic acid	C_16_H_32_O_2_	M − H	255.2337	15.402	2.74	/	3.78	3.96
12	Lauryl hydrogen sulfate	C_12_H_26_O_4_S	M − H	265.1480	9.812	−0.36	/	1.19	ND

“/” indicates that no secondary fragments were detected.

## Data Availability

The original contributions presented in the study are included in the article/Supplementary Materials, further inquiries can be directed to the corresponding authors.

## Supplementary Materials

The following supporting information can be downloaded at: https://www.mdpi.com/article/10.3390/foods14244313/s1, Figure S1: 16S rDNA-based bacterial diversity analysis. Figure S2: UPLC-PDA-MS/MS chromatograms of the white frost on the WF surface. Figure S3: UPLC-PDA-MS/MS chromatograms of the surface powders from NF. Figure S4: UPLC-DAD chromatograms at 284 nm. Table S1: proportion of ECG products with white Frost across different years and batches. Table S2: list of primers used in PCR. Table S3: contents of Mycotoxins in WF and NF samples.
