# Supplementary material for: Characterization of White Frost on Exocarpium Citri Grandis: Flavonoid Crystallization Enhances Anti-Inflammatory Activities
Source: Foods. 2025 Dec 15;14(24):4313. doi: 10.3390/foods14244313 (PMC12732236; doi:10.3390/foods14244313)
Supplement: Supplementary file 1 [file foods-14-04313-s001.zip › foods-4013490-supplementary.pdf]

Table S1 Proportion of ECG products with white Frost across different years and batches.

Date	Manufacturer	Origin	White Frost/Total Number	Proportion of White Frost (%)
2024/04/25	Juxing	Jiangangling	322/1032	31.2
2024/04/26	Juxing	Jiangangling	384/1162	33.0
2024/04/26	Juxing	Pengli	340/1098	31.0
2024/04/27	Juxing	Jiangangling	247/974	24.8
2024/04/28	Juxing	Jiangangling	281/995	28.2
2024/04/29	Juxing	Jiangangling	122/1063	11.5
2024/04/29	Juxing	Pengli	108/1145	9.4
2024/04/30	Juxing	Jiangangling	92/1187	7.7
2025/05/13	Juxing	Feng’eling	128/1184	10.8
2025/05/14	Meihua	Gaofeng	413/1053	39.2
2025/05/13	Meihua	Gaofeng	416/1038	40.1
2025/05/14	Guangdong Agribusiness	Hongfeng	39/1056	3.6

Table S2 List of primers used in PCR.

Primer Name	Primer Sequence (5' to 3')
ITS1-F	CTTGGTCATTTAGAGGAAGTAA
ITS2-R	GCTGCGTTCTTCATCGATGC
338F	ACTCCTACGGGAGGCAGCA
806R	GGACTACHVGGGTWTCTAAT
IL-10-F	CAGTACAGCCGGGAAGACAA
IL-10-R	CCAGCTGGTCCTTTGTTTGA
IL-1β-F	GCTACCTGTGTCTTTCCCGT
IL-1β-R	CGTCACACACCAGCAGGTTA
IL-6-F	TCCAGTTGCCTTCTTGGGAC
IL-6-R	GGTCTGTTGGGAGTGGTATCC
iNOS-F	AAGATGGCCTGGAGGAATGC
iNOS-R	TGCTGTGCTACAGTTCCGAG
NF-κB-F	CCCTACGGAACTGGGCAAAT
NF-κB-R	GCGGAATCGAAATCCCCTCT
TNFα-F	AGCCCACGTCGTAGCAAACCAC
TNFα-R	ACACCCATTCCCTTCACAGAGC
β-actin-F	ATGTGGATCAGCAAGCAGGA
β-actin-R	AAGGGTGTAAAACGCAGCTCA

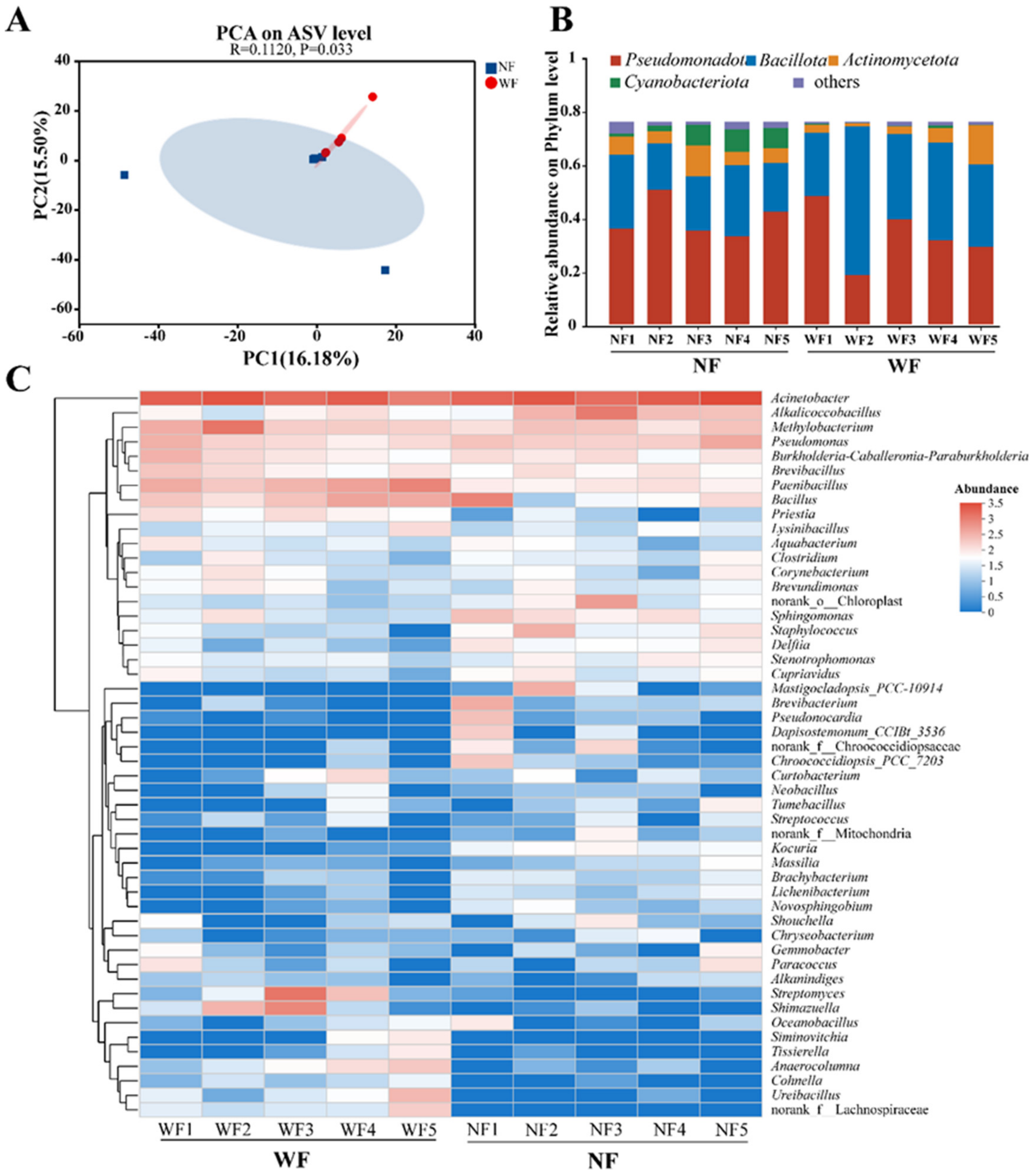

Figure S1 16S rDNA-based bacterial diversity analysis. (A), PCoA at the ASV level based on Bray-Curtis distances; (B), bacterial composition at the genus level; (C), bacterial community composition at the phylum level (top 50 taxa in relative abundance).

Table S3 Contents of Mycotoxins in WF and NF samples

**Analytical Item**	**Method**	**LOQ (μg/kg)**	**WF**	**NF**
Aflatoxin B1	AOAC2008.02-2008	0.1	ND	ND
Aflatoxin B2	AOAC 2008.02-2008	0.1	ND	ND
Total aflatoxins	AOAC 2008.02-2008	0.1	ND	ND
Patulin	AOAC 2000.02-2004	10	ND	ND
Ochratoxin A	AOAC991.44-1996(2002)	1	ND	ND
Zearalenone	AOAC985.18-1988 (2002)	17	ND	ND
Deoxynivalenol	AOAC 986.18-1990	200	ND	ND
Fumonisin B2	AOAC 2001.04-2001	100	ND	ND

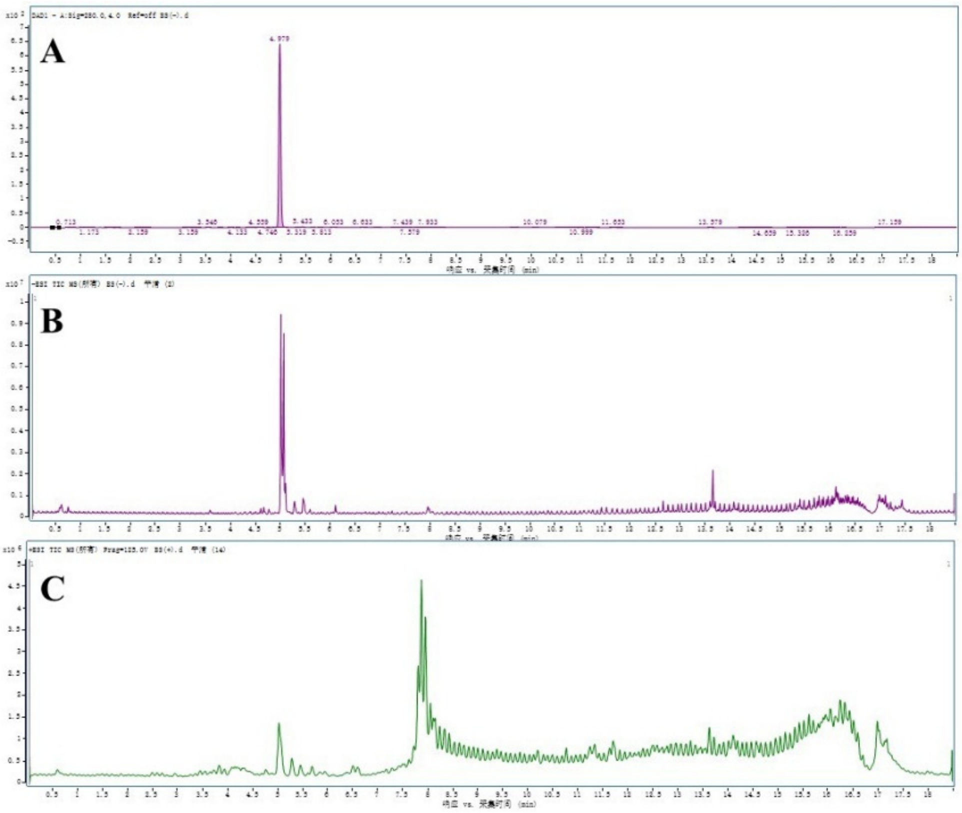

Figure S2 UPLC-PDA-MS/MS chromatograms of the white frost on the WF surface. (A) DAD chromatogram at 284 nm；(B) total ion chromatogram (TIC) in negative ion mode; (C), TIC in positive ion mode.

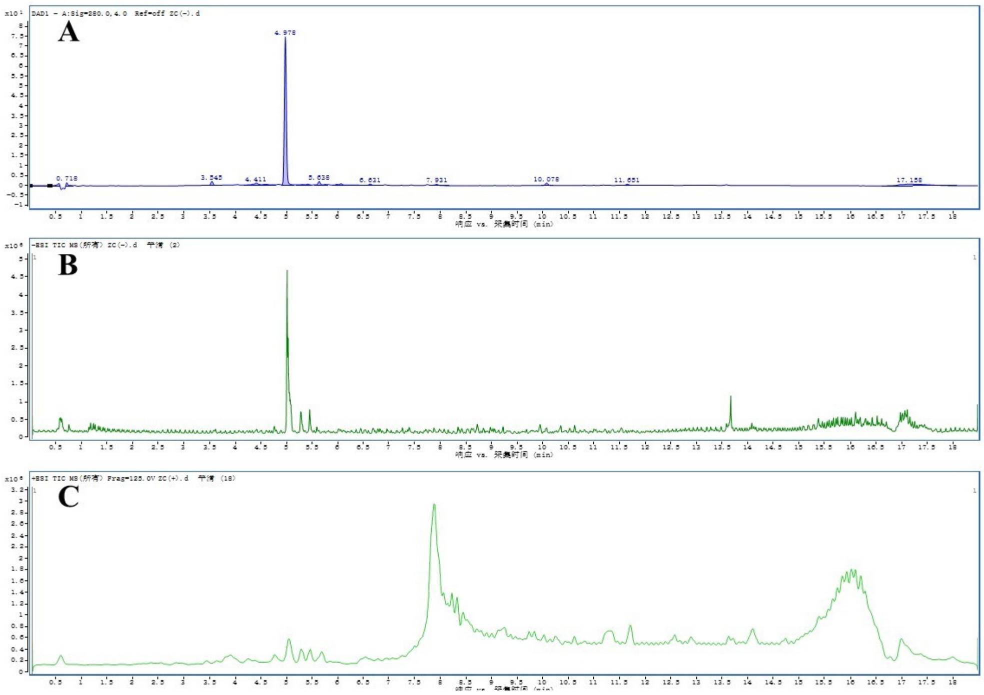

Figure S3 UPLC-PDA-MS/MS chromatograms of the surface powders from NF. (A) DAD chromatogram at 284 nm；(B) TIC in negative ion mode; (C), TIC in positive ion mode.

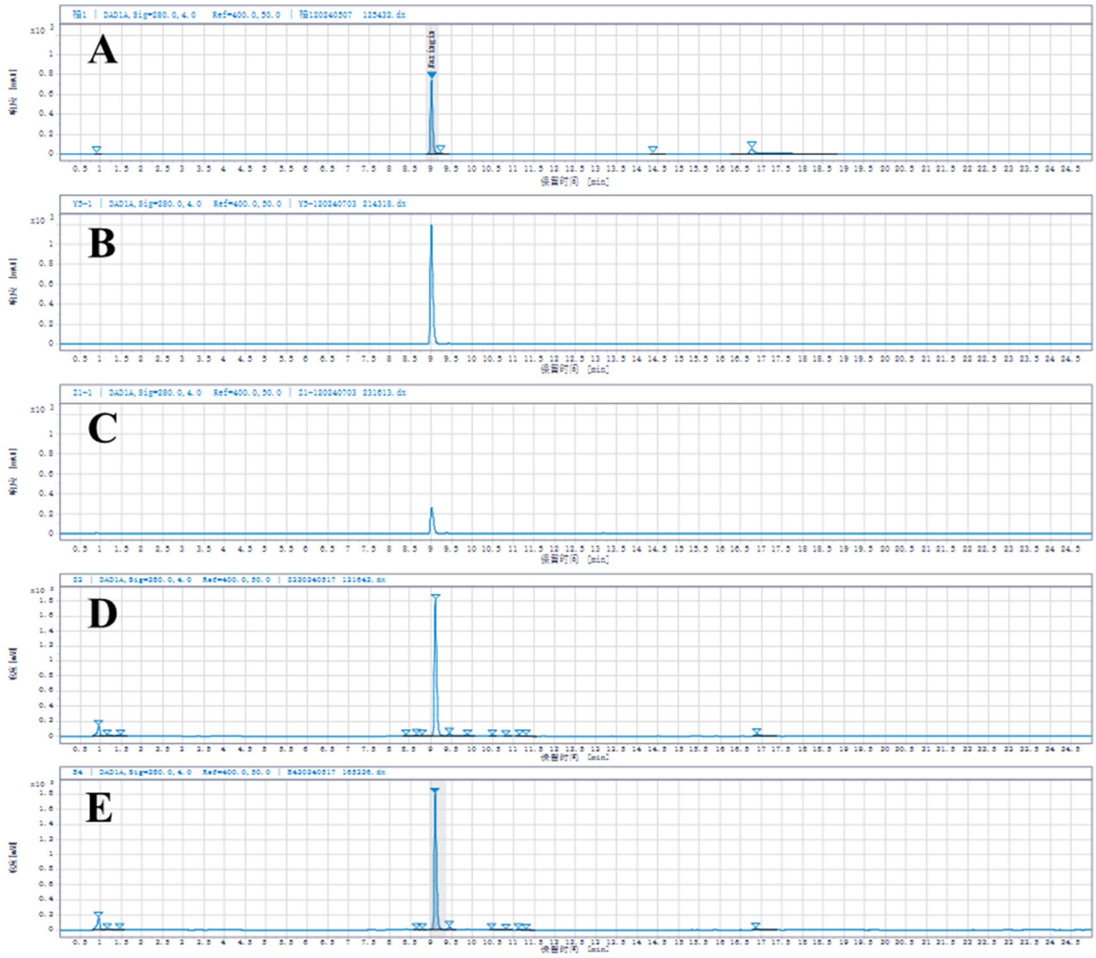

Figure S4 UPLC-DAD chromatograms at 284 nm. (A), naringin standard solution (200 μg/mL); (B–C), surface powders of WF and NF, respectively; (D–F), whole-fruit samples of WF and NF, respectively.
